# Layer-by-layer hybrid chemical doping for high transmittance uniformity in graphene-polymer flexible transparent conductive nanocomposite

**DOI:** 10.1038/s41598-018-28658-6

**Published:** 2018-07-06

**Authors:** Chandan Biswas, Idris Candan, Yazeed Alaskar, Hussam Qasem, Wei Zhang, Adam Z. Stieg, Ya-Hong Xie, Kang L. Wang

**Affiliations:** 1 Department of Electrical Engineering, Center of Excellence for Green Nanotechnologies, University of California, Los Angeles, CA 90095 USA; 2 Department of Materials Science & Engineering, University of California, Los Angeles, CA 90095 USA; 3 California NanoSystems Institute, University of California, Los Angeles, CA 90095 USA; 4 Center for Integrated Nanostructure Physics (CINAP), Institute for Basic Science (IBS), Sungkyunkwan University, Suwon, 16419 Republic of Korea; 5 Department of Physics, Kocaeli University, Izmit, 41380 Turkey; 6 National Center for Nanotechnology, King Abdulaziz City for Science and Technology (KACST), Riyadh, 11442-6086 Saudi Arabia; 7 Maxim Integrated, 160 Rio Robles, San Jose, CA 95134 USA

## Abstract

A traditional transparent conducting film (TCF) such as indium tin oxide (ITO) exhibits poor mechanical flexibility and inconsistent transmittance throughout the UV-VIS-NIR spectrum. Recent TCFs like graphene films exhibit high sheet resistance (R_s_) due to defect induced carrier scattering. Here we show a unique hybrid chemical doping method that results in high transmittance uniformity in a layered graphene-polymer nanocomposite with suppressed defect-induced carrier scattering. This layer-by-layer hybrid chemical doping results in low R_s_ (15 Ω/sq at >90% transmittance) and 3.6% transmittance uniformity (300–1000 nm) compared with graphene (17%), polymer (8%) and ITO (46%) films. The weak localization effect in our nanocomposite was reduced to 0.5%, compared with pristine (4.25%) and doped graphene films (1.2%). Furthermore, negligible R_s_ change (1.2 times compared to 12.6 × 10^3^ times in ITO) and nearly unaltered transmittance spectra were observed up to 24 GPa of applied stress highlighting mechanical flexibility of the nanocomposite film.

## Introduction

Monolayers of carbon atoms packed into a honeycomb lattice in two-dimensional (2D) space (graphene) demonstrate extraordinary electronic and optoelectronic properties^1–4^. Numerous exotic behaviors such as the anomalous quantum Hall effect, the Klein paradox, coherent transport, extraordinarily high mobility, optical modulation, and persistent photoconductance are observed in graphene^5–10^. High quality, flexible transparent conducting films (FTCF) are among the crucial building blocks for optoelectronic technologies ranging from flexible touchscreens to rollable displays, flexible light-emitting devices, and flexible energy conversion applications. Traditional transparent conducting films (TCF) such as indium tin oxide (ITO) are not suitable for flexible electronics technology upgrades due to their poor mechanical flexibility^11–13^ and inconsistent transmittance near the UV-VIS-NIR region^4^. Several nanomaterials such as nanowires^14–17^, conducting polymers^18,19^, metal-polymer layered hybrids^20^, and carbon nanotubes^21^ have shown some potential to replace ITO. Nevertheless, there are numerous challenges in these materials, for example, CNTs and metallic nanowires suffer from long-term instability, uneven surface morphology, and high contact resistance. Conducting polymers and metal hybrids are inadequate due to their large sheet resistance (R_s_).

Graphene is ideally suited as a transparent conduction layer due to extraordinarily high carrier mobility in atomically thin 2D space, transmittance up to 97.6%, and exceptional mechanical flexiblity^5–10,22^. Mechanically exfoliated suspended graphene flake exhibits remarkable mobility (2.3 × 10^5^ cm^2^/V s)^10^. However carrier mobility plummets (2 × 10^3^−2 × 10^4^ cm^2^/Vs) significantly due to the substrate-induced scattering upon transferring graphene layers onto external substrates^23^. Carrier scattering can be reduced in graphene (mobility of 8 × 10^4^ cm^2^/Vs) by transferring it on top of a 2D hexagonal boron nitride (h-BN) or epitaxial ferroelectric gate oxides materials (1.4 × 10^5^ cm^2^/Vs) as demonstrated previously^23,24^. However, most of these results were obtained in nanometer scale devices at low temperatures. Unfortunately, large-scale graphene synthesized by different methods^25^ results in crystalline graphene domains separated by grain boundaries, carbon vacancies, hexagonal lattice defects, and a randomly distributed structural ripple over the surface. These defects, ripples, and grain boundaries, along with substrate induced interfacial trap charges, react as carrier scattering centers and drastically reduce carrier mobility. Numerous adaptations have been made to overcome these challenges for flexible transparent conducting films, such as the use of large area graphene synthesized by chemical vapor deposition (CVD)^26^, interconnected graphene networks^27^, reduced graphene oxide films^28,29^, graphene Langmuir–Blodgett hybrid films^30^ and graphene-polymer nanocomposites^31–33^. However, a comparable TCF performance to that of ITO (R_s_ 10 to 20 Ω/sq at >90% transmittance) has not been achieved to date due to intrinsic defects, ripples, and grain boundary effects in graphene-based nano-carbon materials.

CVD-grown single layer graphene transferred onto SiO_2_ substrates exhibits sheet resistance in the order of few hundred ohms with very high transmittance (around 97%)^26^. Sheet resistance can be reduced to ~30 Ω/sq (at 90% transmittance) by using chemical doping methods^26,34^. However, this value is nearly double that of conventional ITO performance as described above. A novel, comprehensive approach is required that incorporates high quality defect-free large-area graphene, improved graphene stacking, more efficient chemical doping, and non-graphene materials integration to overcome the TCF performance bottleneck.

In this investigation, we demonstrate ITO comparable R_s_ in graphene-polymer nanocomposite that exhibits superior flexibility and transmittance properties. A unique chemical doping method was employed to achieve resistance quenching in the graphene-polymer nanocomposite film while maintaining high optical transparency (15 Ω/sq R_s_ at >90% transmittance). Furthermore, nominal changes in the nanocomposite film transmittance and R_s_ (1.2 times compared to 12.6 × 10^3^ times change in the ITO) were obtained under severe applied mechanical stress up to 24 GPa. These outcomes highlight the strong application potential of novel hybrid nanocomposite materials for highly conductive, transparent and flexible electronics technologies.

## Results and Discussion

**Figure 1:**
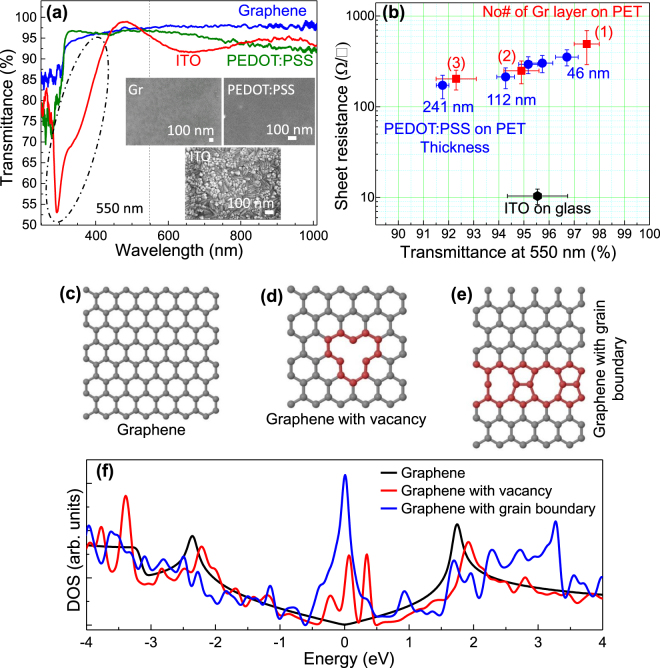
(**a**) Comparison between the transmittance spectra of single layered graphene, 46 nm thick PEDOT:PSS layer, and 200 nm thick ITO film. Figure inset showing the SEM micrographs of graphene (top left), PEDOT:PSS (top right), and the ITO (bottom) film deposited on SiO_2_/Si and glass substrates respectively. (**b**) Transmittance (T) vs. sheet resistance (R_s_) plot for different thicknesses of graphene and PEDOT:PSS films on PET substrate and comparison with ITO film deposited on glass. (**c**–**f**) DOS calculation of graphene (**c**), graphene with carbon vacancy (**d**), and graphene with grain boundary near Dirac point (near zero eV) (**f**).

Conventional ITO thin films exhibit approximately 10 to 20 Ω/sq R_s_ at >90% transmittance. A 200 nm thick ITO thin film typically shows a transmittance spectrum as demonstrated in Fig. 1a. Under these high transmittance conditions (transmittance > 90% at 550 nm, black dotted line in Fig. 1a) ITO films show significantly lower transmittance near the UV-VIS region (highlighted by black dash-dot area) as compared to graphene and PEDOT:PSS films. Transmittance in ITO dramatically decreases in the UV region from 97% (at 450 nm) to 52% (at 300 nm) as highlighted by the dash-dot area. In addition, the rough surface morphology of ITO thin film (Fig. 1a inset) promotes carrier scattering that serves as the key bottleneck for high mobility optoelectronic device applications. In contrast, graphene and conductive polymers such as poly(3,4-ethylene dioxythiophene)-poly(styrene sulfonate) (PEDOT:PSS) show uniform transmittance over a large optical window as demonstrated in Fig. 1a. The transmittance and R_s_ of graphene and PEDOT:PSS thin films (with different thicknesses) transferred on top of polyethylene terephthalate (PET) substrate were compared with ITO performance in Fig. 1b. The transmittance of the PEDOT:PSS films reduces with increasing thickness, from 96.7% (46 nm thick) to nearly 92% (241 nm thick film). R_s_ however, decreases with increasing thickness, from 353 Ω/sq to 172 Ω/sq respectively (see Fig. 1b). High quality single layer graphene used in this study was synthesized by the CVD method^35^. This was verified by Raman spectroscopy (shown in Fig. S1) of single layered graphene with an insignificant D-band, very high G’/G peak ratio of 3.34, and the presence of G* band. This defect-free single layered graphene shows nearly 97.5% transmittance (in accord with previous investigations)^2^ with 490 Ω/sq R_s_. Graphene’s R_s_ can be reduced by multiple layer stacking^34^. However, film transmittance reduces consequently as demonstrated in Fig. 1b. Large-scale single layer graphene synthesized by the CVD method^25^ results in crystalline graphene domains separated by grain boundaries, carbon vacancies, lattice defects and structural ripple distributions randomly over a large area (see Fig. 1c–e). Fig. 1f compares the calculated density of states (DOS) (based on density functional theory^36^, see SI) of pristine graphene (Fig. 1c), graphene with vacancies (Fig. 1d), and graphene with grain boundaries (Fig. 1e). Graphene with vacancies and grain boundaries results in large DOS states near the Dirac point (0.0 eV) compared to pristine graphene. These defects can react as carrier scattering centers, drastically reducing carrier mobility in graphene and serving as the major bottleneck for large area flexible TCF applications.

**Figure 2:**
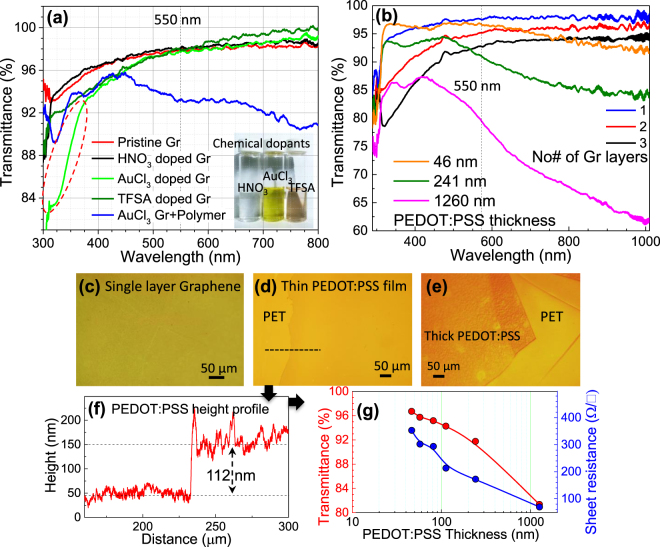
(**a**) Transmittance spectra of single layer chemically doped (using HNO_3_, AuCl_3_, and TFSA dopants) graphene and doped graphene-polymer nanocomposite (incorporating single layered graphene and polymer with different thicknesses). Figure inset represents a photograph of dopant solution dispersed in nitromethane solvent. (**b**) Transmittance spectra of few-layered graphene and PEDOT:PSS polymer film with different thicknesses. (**c**–**e**) Optical micrograph of single layer graphene (**c**), thin (**d**) and thick (**e**) PEDOT:PSS polymer film deposited on PET substrate. (**f**,**g**) Measured thickness profile (**f**) of PEDOT:PSS film on PET substrate and corresponding variations (**g**) in T & R_s_ with film thickness.

Strategies to overcome the conduction bottleneck caused by intrinsic defects and grain boundaries in graphene involve graphene multi-stacking to provide alternative low resistive conduction paths and chemical doping to boost carrier conduction. The unique electronic band structure of graphene allows for modulation of charge carrier conduction and a significant decrease in R_s_ by chemical doping^26,34^. Chemical dopants such as HNO_3_, AuCl_3_, and bis(trifluoromethane)sulfonimide (TFSA) have been previously investigated for graphene films^26,30,33,34^. Here we have compared these doping methods for graphene-polymer nanocomposites and optimized an effective strategy to decrease film R_s_ while maintaining the 90% transmittance for practical applications. A 30 mM dopant concentration was sufficient to achieve R_s_ saturation in previous investigations^37,38^. Therefore, 30 mM dopant (AuCl_3_ and TFSA) concentrations in nitromethane solution were spin coated (4000 rpm for 2 min) on top of the film, followed by hot plate annealing at 80 °C for 10 minutes. Figure 2a compares the transmittance spectra of single layered doped graphene films with pristine graphene and doped graphene-polymer nanocomposite. The transmittance of the HNO_3_, AuCl_3_, and TFSA doped single layered graphene films show similar values near 550 nm (dotted line in Fig. 2a) however they vary slightly around 350 nm. The transmittance of the AuCl_3_ doped single layered graphene dramatically decreases near 300 nm (see red dotted region) which could be due to the formation of a gold nanocluster from Au^0^ and Au^3+^ ions^34,38^. These nanoclusters significantly decreased transmittance in AuCl_3_ doped single layered graphene coated with 46 nm polymer film throughout UV-VIS-NIR region. Figure 2b demonstrates the transmittance spectra of graphene and polymer (PEDOT:PSS) films with different thicknesses transferred on top of PET substrate. Single layer graphene film shows around 97.5% transmittance at 550 nm, similar to the previous investigations^26,34^. Two and three layers of stacked graphene films resulted in a reduction in transmittance of around 94.8% and 92.4% respectively as expected. In comparison, 46 nm, 241 nm, and 1260 nm thick PEDOT:PSS films show transmittance in the range of 96.7%, 91.7%, and 81.3% respectively at 550 nm (see Fig. 2b). It should be noted that transmittance in graphene decreases slightly below 500 nm and transmittance in PEDOT:PSS reduces drastically in NIR regions. R_s_ and transmittance both decrease with increasing thickness of the graphene and PEDOT:PSS films as discussed earlier (Fig. 1b). Figure 2c**–**e shows the optical micrographs of the single layered graphene film, thin (57 nm) and thick (1260 nm) PEDOT:PSS films on PET substrate, respectively. The thickness of the polymer (PEDOT:PSS) thin films was measured by profilometer height profile as demonstrated in Fig. 2f. This enabled us to summarize the variations in transmittance and R_s_ as a function of film thickness as shown in Fig. 2g. Polymer films with different thicknesses were prepared in the range of 46 nm to 1260 nm. Decreasing transmittance and R_s_ were observed with exponential increases in the film thickness. Here, 46 nm film exhibited 96.7% transmittance and 353 Ω/sq R_s_, while 1260 nm film showed 81.3% transmittance and R_s_ decreased to 69 Ω/sq.

**Figure 3:**
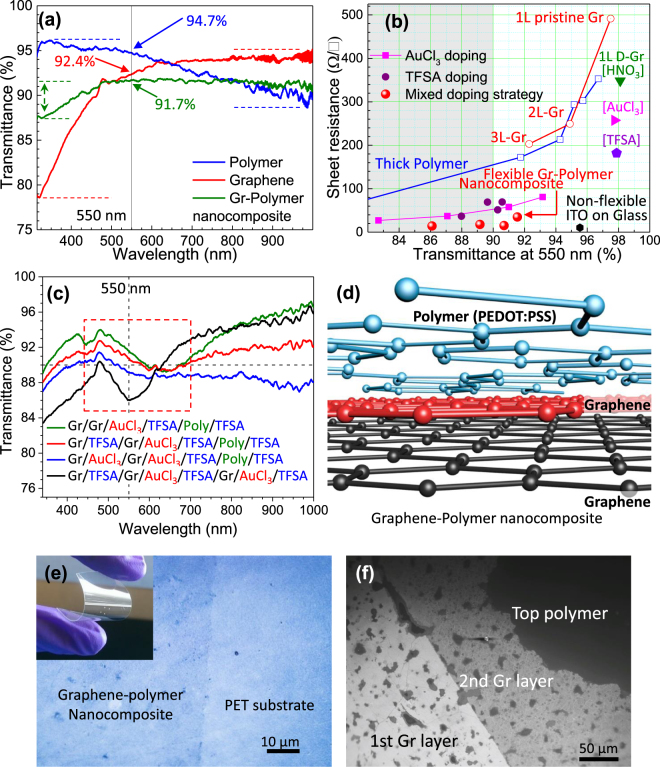
(**a**) Transmittance spectra comparison between three-layered graphene, 100 nm thick polymer and graphene-polymer nanocomposite films. Arrowed markers and dotted lines represent transmittance values at 550 nm and transmittance maximum-minimum respectively. (**b**) T vs. R_s_ graph of the different types of transparent conducting films investigated in this study, including pristine and doped graphene films, polymer with different thicknesses, single and hybrid doped films, and graphene-polymer nanocomposite. (**c**) Transmittance spectra of graphene-polymer nanocomposite with different layered structure. (**d**) Schematic model of the graphene-polymer nanocomposite film. (**e**) Optical micrograph of the graphene-polymer nanocomposite film on PET substrate. (**f**) SEM micrograph of the nanocomposite film shows individual graphene and polymer layers in the nanocomposite stacking.

One of the major advantages in the above graphene-polymer nanocomposite is the transmittance uniformity over a broad wavelength range. Graphene and PEDOT:PSS (polymer) both exhibit irregular transmittance spectra over the spectral window between 300 nm to 1000 nm (see Fig. 3a). Graphene films exhibit high transmittance near NIR however, the transmittance decreases drastically at low wavelength regions starting from 500 nm. 92.4% transmittance (at 550 nm) was observed in three-layered graphene films; however, it shows maximum (95% transmittance at 1000 nm) and minimum (79% transmittance at 320 nm) transmittance in NIR and UV regions respectively. This suggests that nearly 17% transmittance variation exists in few layered graphene films. The transmittance variation in polymer thin film (100 nm thick) was obtained at around 8% showing maximum (96% transmittance at 345 nm) and minimum (88% transmittance at 987 nm) in the UV and NIR regions respectively. In comparison, transmittance variation was significantly reduced to 4.4% in graphene-polymer nanocomposite films maximizing transmittance (91.7%) around the 550 nm wavelength, ideally suited for large number of optoelectronic applications.

Figure 3b summarizes the different types of transparent conducting films optimized in this work, incorporating numerous graphene and polymer films with different thicknesses, various chemical doping methods, and comparisons with conventional ITO. Sheet resistance and transmittance decrease with increasing film thickness in graphene and polymer thin films as discussed earlier. The film thickness adequate for 90% transmittance resulted in around 200 Ω/sq sheet resistance both in few-layered graphene and PEDOT:PSS film. Sheet resistance can be reduced by chemical doping as demonstrated. The sheet resistance was reduced in single layered graphene from 490 Ω/sq to 348 Ω/sq, 257 Ω/sq, and 181 Ω/sq without altering transmittance much using the HNO_3_, AuCl_3_, and TFSA doping methods respectively. It should be noted that the single layered graphene doped by HNO_3_ results in marginally higher transmittance of 98.1% compared to pristine graphene; this could be due to the etching and defect generated by the strong HNO_3_ acid solution^26,34^. In comparison, AuCl_3_ doping does not increase defects in graphene however, resulted in aggregation of gold Au^0^ and Au^3+^ ions on the film surface^34,38^. These aggregated nanoparticles scatter with incident light and reduce film transmittance. Different polymer film thicknesses were used on top of the chemically doped graphene films to further decrease R_s_. Different thicknesses of the graphene-polymer nanocomposite in the graphene/AuCl_3_/graphene/AuCl_3_/polymer structures (“AuCl_3_ doping” legend in Fig. 3b) were prepared by changing polymer thickness from 46 nm to 100 nm. R_s_ can be reduced from 81 Ω/sq to 27 Ω/sq using this method, however, the transmittance of the sample reduces drastically from 93% to 82.6% respectively. This could be due to the gold nanoparticle formation in the film as described earlier. This suggests that the AuCl_3_ doping method results low sheet resistance however, it faces major disadvantage due to a considerable decrease in the film transmittance below 90% (grey shaded region in Fig. 3b). In comparison, the TFSA doping method reduces R_s_ to a certain extent without compromising transmittance (see “TFSA doping” legend Fig. 3b). The TFSA doped graphene polymer nanocomposite in the graphene/TFSA/graphene/TFSA/polymer structure was compared with the AuCl_3_ doping method using similar polymer thickness variations (46 nm to 100 nm). TFSA doped nanocomposite with 90.6% transmittance results in R_s_ around 69 Ω/sq. R_s_ can be further reduced up to 37 Ω/sq by increasing nanocomposite thickness; however, transmittance reduces to less than 90% (88%). It is evident from the Fig. 3b that the TFSA doping method is beneficial for decreasing electrical R_s_ without compromising much of the optical transmittance in nanocomposite films compared to the AuCl_3_ doping method. Therefore, a novel hybrid chemical doping method was developed, incorporating TFSA doping (advantageous for optical transmittance) and AuCl_3_ doping (beneficial for lower R_s_) respectively. This unique doping strategy enabled us to achieve R_s_ around 15 Ω/sq with 90.7% transmittance in the graphene/TFSA/graphene/AuCl_3_/TFSA/polymer/TFSA nanocomposite structure (“mixed doping strategy” legend). Increasing the nanocomposite film thickness from this point could decrease R_s_ further (14 Ω/sq as shown in Fig. 3b), however transmittance would reduce drastically to less than 90% (86%), making it undesirable for numerous optoelectronic applications (see Table S1).

The transmittance spectra of hybrid doping methods are compared in Fig. 3c. Two layered doped graphene is stacked together with 57 nm thick PEDOT:PSS film on top (see schematic in Fig. 3d) in the graphene-polymer nanocomposite to optimize lowest R_s_ while keeping >90% transmittance. Single use of AuCl_3_ doping in the graphene/graphene/AuCl_3_/TFSA/polymer/TFSA nanocomposite structure results in high transmittance (91.5%) due to less nanocluster formation from Au^0^ and Au^3+^ ions, however R_s_ was not considerably lowered (35 Ω/sq). Moreover, the transmittance variation in this structure was obtained around 8.4%. TFSA doping was introduced between graphene layers while keeping other layers identical (in the graphene/TFSA/graphene/AuCl_3_/TFSA/polymer/TFSA nanocomposite structure) in order to decrease the sheet resistance further without compromising transmittance below 90% (see Fig. 3c). Encouraging resistance quenching was obtained as R_s_ decreased to 15 Ω/sq and transmittance around 90.7%, comparable with ITO (Fig. 3b). This result suggests that the sheet resistance in the doped nanocomposite can be significantly quenched compared with pristine graphene and polymer films. Furthermore, transmittance variations are significantly reduced to 3.6% in this structure, resulting in a maximum (92.7% transmittance at 480 nm) and minimum (89.3% transmittance at 650 nm) both in the visible wavelength regions (see dotted region in Fig. 3c). Low R_s_ (comparable to ITO), high transmittance (>90%), low transmittance variations (<4%), and transmittance maximum/minimum in the visible region demonstrate the advantages of this nanocomposite structure. Two time use of AuCl_3_ doping in the graphene/AuCl_3_/graphene/AuCl_3_/TFSA/polymer/TFSA nanocomposite structure resulted in comparable R_s_ (17.8 Ω/sq) however, transmittance reduced below 90% (89.1%). Three-layered graphene in the nanocomposite (graphene/TFSA/graphene/AuCl_3_/TFSA/graphene/AuCl_3_/polymer/TFSA) resulted in significant reduction in film transmittance up to 86% and resulted in similar R_s_ (14 Ω/sq). An optical micrograph of the chemically doped graphene-polymer nanocomposite on PET substrate is shown in Fig. 3e (digital image in the inset). The scanning electron micrograph (SEM) demonstrated in Fig. 3f shows first the doped graphene layer (from left) followed by a second doped graphene layer (middle) and the doped polymer layer (right) of the nanocomposite film. Clear grain boundaries were observed in the first and second graphene layers (see Fig. S2). These grain boundaries react as a scattering center during carrier conduction and reduce sheet resistance as demonstrated earlier. These grain boundaries were further covered by a polymer layer on top, as shown in Fig. S2. The surface morphology of the nanocomposite film was comparably smoother compared to graphene regions. This could be beneficial for the surface roughness of sensitive optoelectronic devices where surface roughness plays crucial role in device performance.

**Figure 4:**
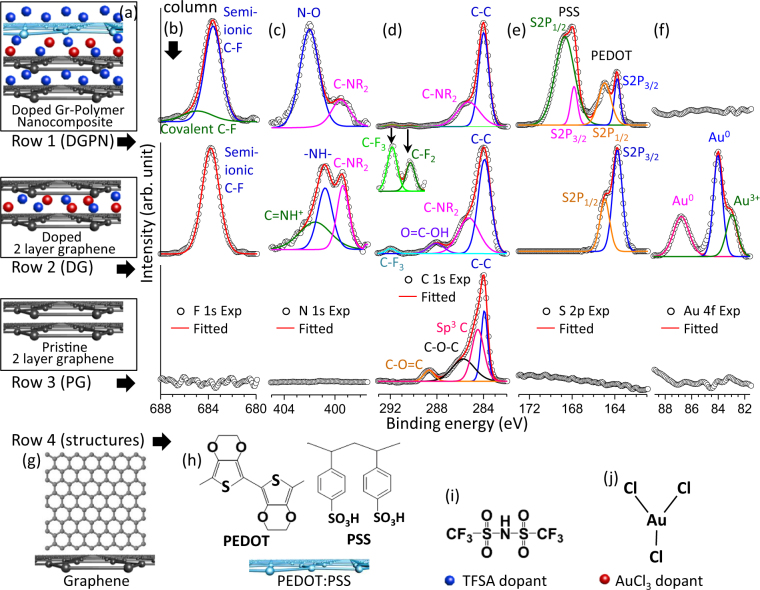
(**a**) Schematic models of the DGPN, DG, and PG structures in column (**a**). (**b**–**f**) Comparison between XPS spectra in F 1s (**b**), N 1s (**c**), C 1s (**d**), S 2s (**e**), Au 4f (**f**) regions of DGPN, DG, and PG structures respectively. (**g**) Schematic model of the single layer pristine graphene. (**h**) Chemical structure (top) and schematic model (bottom) of the PEDOT:PSS polymer. (**i**) Chemical structure of the TFSA dopant molecule. (**j**) Chemical structure of the AuCl_3_ dopant molecule.

Charge transfer during the chemical doping process can be monitored using X-ray photoelectron spectroscopy (XPS). Figure 4 compares the XPS spectra from doped graphene-polymer nanocomposite (DGPN, row 1 in Fig. 4), doped two-layered graphene films (DG, row 2 in Fig. 4), and pristine two-layered graphene (PG, row 3 in Fig. 4) films. The hybrid chemical doping method contained both TFSA and AuCl_3_ doping as described earlier. TFSA dopant solution consists of fluorine, nitrogen, and sulfur atoms^37,38^, whereas gold ions were observed previously in AuCl_3_ dopants^34^. Therefore, F1s, N1s, C1s, S2p, and Au4f XPS spectra were compared from higher to lower binding energies respectively (column b to f in Fig. 4). Characteristic peaks from carbon-fluorine (C-F) bonds (ionic and covalent) can be observed in F1s and C1s spectra^39^. Strong semi-ionic C-F bonds from TFSA were observed in DGPN and DG samples near 683.6 eV. In addition, the covalent C-F bond was deconvoluted in the F1S spectrum near 685 eV. Furthermore, C1S spectra also exhibit C-F bonds from TFSA doping as demonstrated in Fig. 4d. DGPN shows distinct C-F_3_ and C-F_2_ bonds near 291.8 eV and 290.3 eV respectively. In comparison, DG samples only result in C-F_3_ bonds near 291.8 eV. These C-F bond locations from TFSA doping agreed adequately with previous investigation^38,39^. Strong carbon-nitrogen (C-N) peaks were observed in DGPN and DG samples as shown in Fig. 4c. The presence of amine (C-NR_2_) peaks verified TFSA doping in DGPN and DG samples both for N1s and C1s spectra near 399.4 eV and 285.3 eV respectively. Moreover, nitrogen related bonds such as N-O (402 eV), -NH- (400.8 eV), and C = NH^+^ (401.6 eV) were observed in the N1s deconvoluted spectrum and confirmed TFSA doping in DGPN & DG samples respectively (see Fig. 4c). Furthermore, the sulfur atoms (Fig. 4h) of used PEDOT:PSS in DGPN exhibited clear S2P_1/2_ (164.9 eV); S2P_3/2_ (163.8 eV) peaks form PEDOT; and S2P_1/2_ (168.6 eV), S2P_3/2_ (167.8 eV) peaks form PSS, similar to previous investigations^40^. Sulfur related peaks from TFSA doping were also found in the DG sample, as shown in row 2 of Fig. 4e (S2P_1/2_ at 164.9 eV, and S2P_3/2_ at 163.8 eV). All of this evidence suggests strong TFSA doping in DGPN and DG samples compared to pristine graphene, where no fluorine, nitrogen, and sulfur-related peaks were observed (see row 3 of Fig. 4b,e). AuCl_3_ doping in the DG sample resulted in strong Au^0^ (near 86.7 eV and 83.9 eV) and Au^3+^ (near 82.9 eV) related peaks in the Au 4f XPS spectrum, similar to the previous investigations^34^. Although AuCl_3_ doping was used in DGPN sample however, the doping layer was covered with two-fold TFSA dopant layers and the 57 nm thick polymer layer; this could be the reason for the absence of gold-related peaks in Au 4f XPS spectrum. It should be noted that all of the samples showed strong C-C peaks (near 284 eV) from graphene however, PG samples showed conventional Sp^3^ carbon (nearly 284.4 eV), C-O-C (285.6 eV), and C-O=C (288.6 eV) related peaks in pristine graphene similar to the previous investigations^4,25,39^ (see row 3 of Fig. 4d). These results clearly highlight the significance of this chemical doping technique for interlayer ionic/covalent bond formation in the nanocomposite. No covalent or ionic bonds were found in the stacked multilayer films without hybrid chemical doping. However, the formation of strong semi-ionic and/or covalent C-F, C-F_2_, C-F_3_, C=NH^+^, N-O, -NH-, and sulfur-related bonds were observed between the stacked multilayers by introducing this simple layer-by-layer hybrid chemical doping method. These chemically induced bonds could result improved structural integrity (further investigation required) and optoelectronic nanocomposite properties as demonstrated in this study.

**Figure 5:**
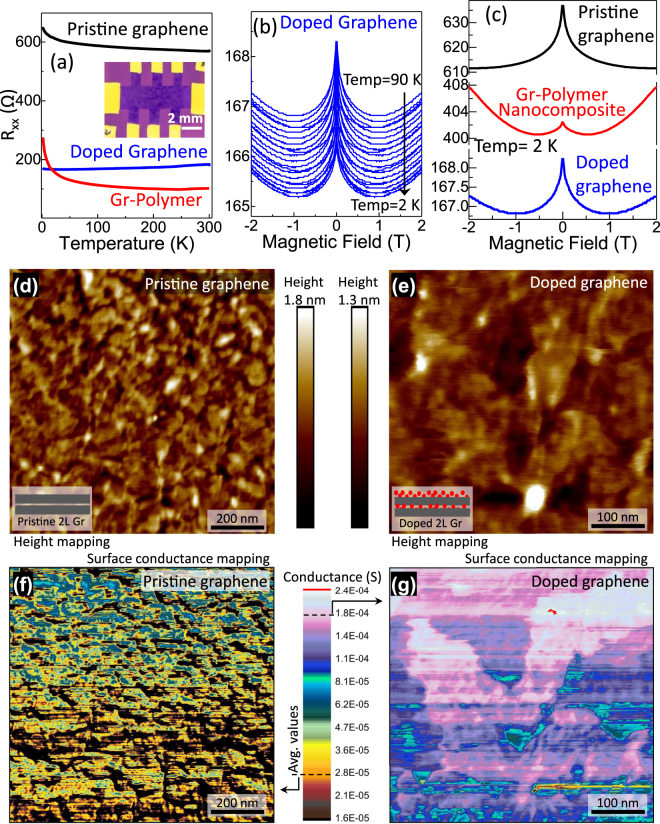
(**a**) Variations in longitudinal resistance (R_xx_) with temperature (300 K to 2 K) without magnetic field for DGPN, DG, and PG device structures. The inset shows the optical micrograph of the Hall-bar device geometry on SiO_2_/Si substrate. (**b**) Variations in R_xx_ with applied magnetic field (over ±2 tesla) in DG device under the temperature range from 2 K to 90 K. (**c**) Comparison of R_xx_ and week localization effect (R_xx_ near B = 0) at with applied magnetic field (over ± 2 tesla) for DGPN, DG, and PG device structures at 2 K. (**d**–**g**) AFM micrograph of the two-layered pristine graphene (**d**) and chemically doped two layered graphene film (**e**) prepared using hybrid doping method. (**f**,**g**) *In-situ* surface conductance mapping of the same measurement as described above (pristine graphene (**d**), doped graphene film (**e**)). 5 mV applied voltage and 49 nN applied force were kept constant during the measurements and identical color scale was employed for direct comparison.

It is evident from the above investigations that graphene-polymer layered nanocomposites exhibit improved optoelectronic properties compared to its close counterparts such as graphene, polymer, and ITO films for FTCF applications. In addition, low-temperature transport measurements were conducted by pattering DGPN, DG, and PG samples in Hall-bar geometry on SiO_2_/Si substrate (see inset of Fig. 5a). Figure 5a shows variations in longitudinal resistance (R_xx_) in the temperature range from 300 K to 2 K in the absence of a magnetic field. At 300 K, pristine graphene (PG) devices showed R_xx_ close to 570 Ω, whereas R_xx_ reduced to 200 Ω in DG due to chemical doping. Further resistance quenching was observed (102 Ω) in nanocomposite (DGPN), similar to the observed R_s_ trends. At low temperature (2 K), the R_xx_ value increased rapidly in PG (from 570 Ω to 647 Ω) and increased exponentially in DGPN (from 102 Ω to 270 Ω). In contrast, the value of R_xx_ dropped in DG (from 200 Ω to 168 Ω). These low temperature rapid R_xx_ increases in DGPN and PG could be attributed to mesoscopic resistance fluctuation effects near the neutrality point in graphene^41^. Furthermore, the mesoscopic resistance fluctuation effects are related to the increasing phase coherence length known as the weak localization effect in graphene^42^. This weak localization effect can be further confirmed by the sharp R_xx_ increase near zero magnetic field (B = 0). Figure 5b demonstrates the variations in R_xx_ with an applied magnetic field in the DG sample in the temperature range from 2 K to 90 K. The weak localization peak observed in R_xx_ near B = 0 resulted from electronic coherent backscattering effects similar to previous reports^41,42^. All of the samples (DGPN, DG, PG) show weak localization peaks in R_xx_ near B = 0 at 2 K temperature (see Fig. 5c) however, their absolute R_xx_ values and peak height vary drastically. Absolute R_xx_ (over ± 2 tesla) was similar to the film resistance as discussed in Fig. 5a. The highest R_xx_ was observed in PG (~637 Ω at B = 0), followed by DGPN (~402 Ω at B = 0), and DG (168 Ω at B = 0). One of the intriguing facts to be noted here is that the carrier coherent backscattering caused by the weak localization effect was significantly reduced in the doped graphene-polymer nanocomposite (DGPN) sample. This was manifested by the reduction of weak localization peak height (at B = 0) in DGPN: up to 0.5% compared to 4.25% in PG and 1.2% in DG samples. These results strongly suggest that the reduction in carrier scattering and consequent resistance quenching in DGPN could be due to the reduction of grain boundaries, carbon vacancies, lattice defects and structural ripple-related carrier scattering processes.

The above investigation suggests improved carrier conduction in doped graphene and doped nanocomposite samples as compared to that in pristine graphene. Consequently, surface conductance of the chemically doped samples could be higher than pristine graphene. Figure 5d–g compares the surface conductance between pristine graphene (PG) and chemically doped graphene (DG) samples (prepared using the hybrid doping method described earlier). Figure 5d,e represent conductive atomic force microscope (AFM) height profile images for PG and DG samples respectively. Corresponding surface conductance mapping images are presented in Fig. 5f,g respectively. The applied voltage (5 mV) and force (49 nN) induced from AFM tip were kept constant during the conductive AFM measurements and data were plotted in the same color scale to enable direct comparison. The average surface conductance observed in the PG was in the order of 2.78 × 10^−5^ S (see dotted line at the bottom of the color scale). In comparison, the chemically doped graphene sample exhibits surface conductance one order of magnitude higher in the range of 1.79 × 10^−4^ S (see dotted line at the top of the color scale in Fig. 5f,g). The current-voltage characteristics (Fig. S3) measured between the AFM tip and film surface clearly demonstrate higher current flow in the DG compared to the PG sample. This indicates that surface conductance in the graphene films can be increased by using the hybrid chemical doping method. The surface conductance properties of the DGPN could not be compared directly with PG and DG due to the mechanically soft polymer coating on top surface. In this case, that applied force was restricted to 28.2 nN in order to prevent mechanical damage to the polymer layer. The AFM height profile map and surface current plot (see Fig. S4) shows measured current in the order of 4 nA range in DGPN sample.

**Figure 6:**
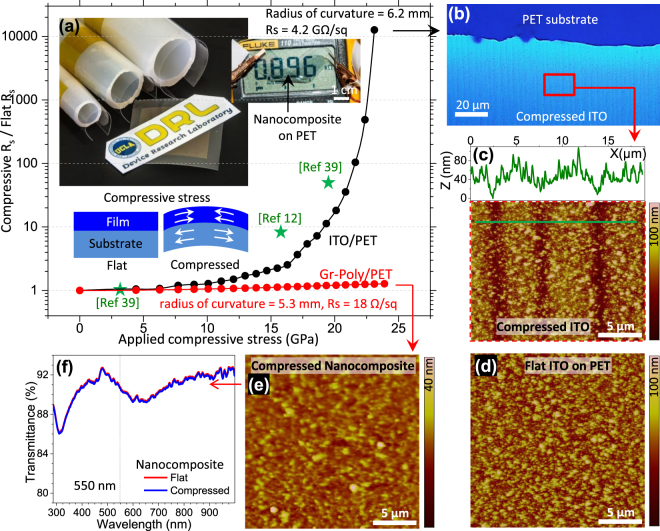
(**a**) Changes in the film sheet resistance (final compressive R_s_/initial flat R_s_) under applied compressive stress up to 23 GPa in 100 nm thick ITO film and DGPN on a flexible PET substrate. Samples were rolled up in different curvature using cylindrical tubes (top left inset) to apply fixed compressing stress on the attached films. Photograph of the wrinkled conducting graphene-polymer nanocomposite film on PET substrate (top right inset). Bottom inset representing the schematic illustration of the flat and compressed thin film on a flexible substrate. (**b**) Optical micrograph of the compressed 100 nm ITO film on PET substrate (under 23 GPa compressive stress) showing clear mechanical crack lines. (**c**,**d**) AFM micrograph of the same sample (**b**) showing ITO crack height profile (**c**) compare to the flat ITO morphology on PET substrate without stress (**d**). (**e**) AFM micrograph of the graphene-polymer nanocomposite film under 24 GPa applied stress on PET substrate. (**f**) Transmittance spectra comparison between compressed (24 GPa stress) and flat (without applied stress) graphene-polymer nanocomposite on PET substrate.

Poor mechanical flexibility of thin film ITO is among its major disadvantages for flexible electronics applications. Significant resistance changes occur in ITO films under stress. Figure 6a compares changes in the film sheet resistance (final compressive R_s_/initial flat R_s_) under applied compressive stress up to 23 GPa in a 100 nm thick ITO film and DGPN on a flexible PET substrate. Initially, ITO and DGPN films demonstrated approximately 10 and 15 Ω/sq sheet resistances respectively. The change in ITO sheet resistance remained small (1.07 times) up to very low applied compressive stress (6 GPa) whereas it increased sharply up to 12.6 × 10^3^ times at 23 GPa. In this stage the ITO film demonstrated a very high R_s_ value of 4.2 GΩ/sq, suggestive of significant damage to the film. Previous investigations observed similar resistance increments in ITO film under applied stress (see reference data points in Fig. 6a)^12,43^. Mechanical cracks were clearly observed under optical micrographs (see Fig. 6b) and AFM micrographs (Fig. 6c), with nearly 80 nm crack depth under 23 GPa applied compressive stress. This was compared with ITO film deposited on a flat PET substrate (Fig. 6d) without any applied stress in which no cracks were observed. In comparison, the nanocomposite film shows only 1.2 times increase in sheet resistance under applied stress up to 24 GPa. AFM morphology of the compressed DGPN (under 24 GPa applied stress) did not reveal any mechanical crack formation as shown in Fig. 6e. Furthermore, the compressed DGPN sample shows a nearly identical transmittance spectrum compared to flat DGPN without applied stress (see Fig. 6f). These results highlight the very high mechanical stability of graphene-polymer nanocomposite films with a nearly unchanged transmittance spectrum and nearly unaltered sheet resistance when subject to large (24 GPa) applied mechanical stress. Previous investigations demonstrated high electro-mechanical stability in graphene and polymer films compared to inorganic ITO films^4,34^. However, defects, ripples and grain boundaries in the film react as carrier scattering centers and are vulnerable to high applied mechanical stress^4^. The results above demonstrate that the multilayer conductive stacking and hybrid chemical doping strategy can maintain unaltered carrier conduction under high mechanical stress.

## Conclusion

In summary, we have demonstrated a novel graphene-polymer flexible transparent conducting nanocomposite that suppresses carrier scattering induced by graphene grain boundaries, carbon vacancies, lattice defects and structural ripple in graphene films. Unique hybrid chemical doping methods incorporating different dopant species were employed for resistance quenching (15 Ω/sq) while maintaining high optical transparency (>90% transmittance at 550 nm) in nanocomposite films (comparable to ITO and lowest so far among carbon-based FTCF). Moreover, the nanocomposite shows tremendous transmittance uniformity (3.6%) in the VIS-NIR range (300 nm to 1000 nm) compared to graphene (17% in three-layered graphene film), polymer (8% in 100 nm thick) and ITO (46% in 200 nm thick) films. Furthermore, the carrier coherent backscattering and consequent resistance quenching in the nanocomposite were significantly reduced (manifested by the weak localization peak height at B = 0): up to 0.5% compared to 4.25% in pristine graphene and 1.2% in doped graphene films. Moreover, the nanocomposite film shows a nearly identical transmittance spectrum and negligible resistance change (1.2 times) under 24 GPa applied stress compared to 12.6 × 10^3^ times resistance change in the ITO. These results clearly highlight the strong practical application potentials of novel hybrid nanocomposite materials for highly conductive, transparent and flexible electronics and could serve as a realistic replacement of conventional ITO, graphene, and polymer films.

## Methods

Single layered graphene was synthesized using a low-pressure chemical vapor deposition (LP-CVD) system on 100 µm thick Cu foil. First, Cu foil was annealed for 5 hrs at 1020 °C under a 145 sccm argon and 29 sccm hydrogen gas mixture followed by chemical polishing using Cu etchant solution (CE-100, Transene Company, Inc.). Graphene growth was conducted at 1000 °C for 30 min under 500 mTorr with a 113 sccm methane and 12 sccm hydrogen gas mixture. The graphene layer was transferred onto the target substrate using the polymethyl methacrylate (PMMA) thin film and deionized water method. Commercially available PMMA (Sigma Aldrich) solution was spin coated on top of the graphene/copper foil with 700 rpm for 7 seconds followed by 1500 rpm for 60 seconds. This was floated on top of the commercially available Cu etchant solution (CE100) for 30 min and deionized water was used to wash etchant residues. Subsequently, the Gr/PMMA films were transferred to the target substrates and acetone was used to remove PMMA layers. 30 mM TFSA and AuCl_3_ dopant was dispersed in nitromethane (Sigma Aldrich) and the supernatant was used as a spin coating solution (4000 rpm for 2 min followed by hot plate annealing at 80 °C for 10 minutes) to implement chemical doping. High conductive PEDOT:PSS polymer solution (HIL-1005 from Sigma Aldrich) was spin coated on top of the doped graphene layers for graphene-polymer nanocomposite synthesis. Spin coating speed (from 1000 rpm to 6000 rpm for 60 sec) was controlled to achieve desired PEDOT:PSS thickness (from 46 nm to 1260 nm thick films respectively). Each nanocomposite layer was annealed at 80 °C for 10 min under atmospheric conditions during the synthesis process. A 30 watt oxygen plasma was used for 20 min for Hall-bar device patterning of the nanocomposite layers. 5 nm Cr and 100 nm Au thin films were deposited using e-beam metal evaporation process for device contacts. The sheet resistance of the fabricated samples was measured by four probe Van-Der-Pauw technique using a semiconductor characterization system (Keithley 4200). Commercially available ITO coated glass was used for transmittance and sheet resistance comparisons. A 100 nm thin ITO film was deposited on PET substrate for compressive stress comparison using RF sputtering system. AFM measurements were conducted using an SPA 300 HV, Seiko Instrument Inc. system. Field emission scanning electron micrographs were obtained from a JEOL JSM-6700F FE-SEM system. Transmittance spectra were obtained by using a UV–Vis spectroscopy system (Ocean Optics).

## Electronic supplementary material

Supplementary Information
