# Supplementary material for: Layer-by-layer hybrid chemical doping for high transmittance uniformity in graphene-polymer flexible transparent conductive nanocomposite
Source: Sci Rep. 2018 Jul 6;8:10259. doi: 10.1038/s41598-018-28658-6 (PMC6035180; doi:10.1038/s41598-018-28658-6)
Supplement: Supplementary file 1 — Supplementary Information [file 41598_2018_28658_MOESM1_ESM.docx]

**Supplementary Information**

Layer-by-layer hybrid chemical doping for high transmittance uniformity in graphene-polymer flexible transparent conductive nanocomposite

Chandan Biswas,*^,†,§^ Idris Candan,^†,⊥^ Yazeed Alaskar,^†,⁋^ Hussam Qasem,^†^ Wei Zhang,^‡,₸^ Adam Z. Stieg,^∇^ Ya-Hong Xie^‡^ and Kang L. Wang*^,†^

^†^Department of Electrical Engineering, Center of Excellence for Green Nanotechnologies, ^‡^Department of Materials Science & Engineering, ^∇^California NanoSystems Institute, University of California, Los Angeles, CA 90095, USA.

^§^Center for Integrated Nanostructure Physics (CINAP), Institute for Basic Science (IBS), Sungkyunkwan University, Suwon 16419, Republic of Korea.

^⊥^Department of Physics, Kocaeli University, Izmit 41380, Turkey.

^⁋^ National Center for Nanotechnology, King Abdulaziz City for Science and Technology (KACST), Riyadh 11442-6086, Saudi Arabia.

^₸^Maxim Integrated, 160 Rio Robles, San Jose, CA 95134, USA

**DFT calculation details:**

Our calculations are based on density functional theory (DFT) as implemented in the Fritz Haber Institute ab initio molecular simulations package (FHI-AIMS). [1] This is an all-electron full potential DFT code that uses numeric atom centered orbitals as its basis set. We use the Perdew-Burke-Ernzerhof (PBE) approximation of the generalized gradient approximation (GGA) for the exchange-correlation functional. [2] We used the Tkatchenko-Scheffler method [3] to include “Van der Waals” interactions in the DFT calculations. To model the defective graphene, we use graphene supercells of different sizes with a vacuum region of 50 Å in the z-direction. A 4x 4x 1 k-point grid is used for the self-consistent field (s.c.f) calculations while 40x 40x 10 k-point grid is used for the density of states calculations. The convergence of the results has been carefully tested with respect to the system size, the basis set, and the density of the numerical integration mesh.

**References:**

[1] V. Blum, R. Gehrke, F. Hanke, P. Havu, V. Havu, X. Ren, K. Reuter, and M. Schefﬂer, Comput. Phys. Commun. 180, 2175 (2009).

[2] J. P. Perdew, K. Burke, and M. Ernzerhof, Phys. Rev. Lett. 77, 3865 (1996).

[3] A. Tkatchenko and M. Schefﬂer, Phys. Rev. Lett. 102, 073005 (2009).


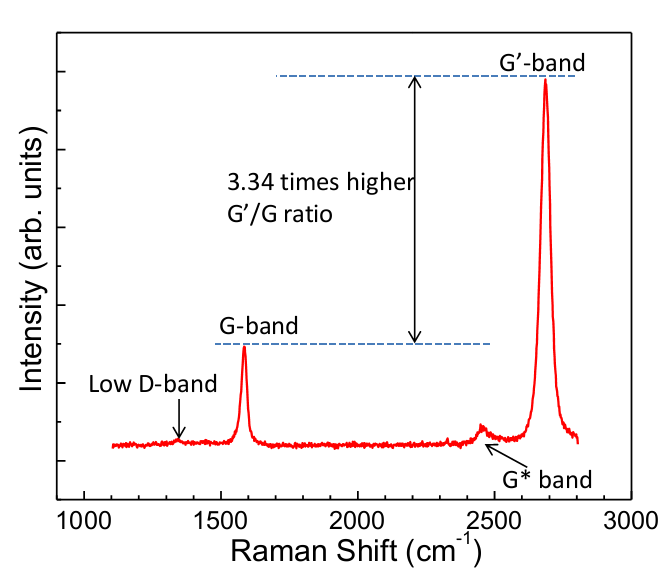


**Figure S1:** Raman spectrum of single layered graphene synthesized using low pressure chemical vapor deposited (LP-CVD) system on 100 µm thick Cu foil. Prior to the Raman measurement graphene layered was transferred on top of a 300 nm thick SiO_2_/Si substrate.


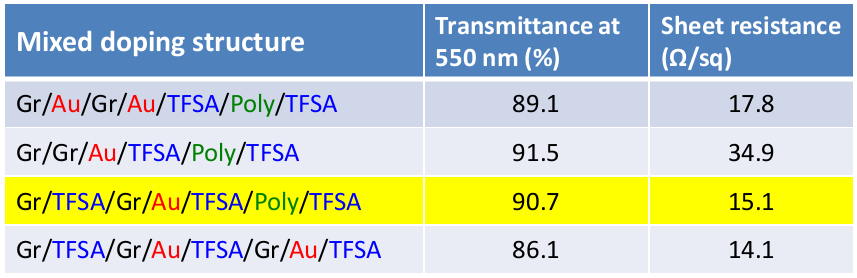


**Table S1:** Graphene-polymer flexible conducting layered nanocomposite with different mixed doping structure and their corresponding transmittance (at 550 nm) and sheet resistance values.


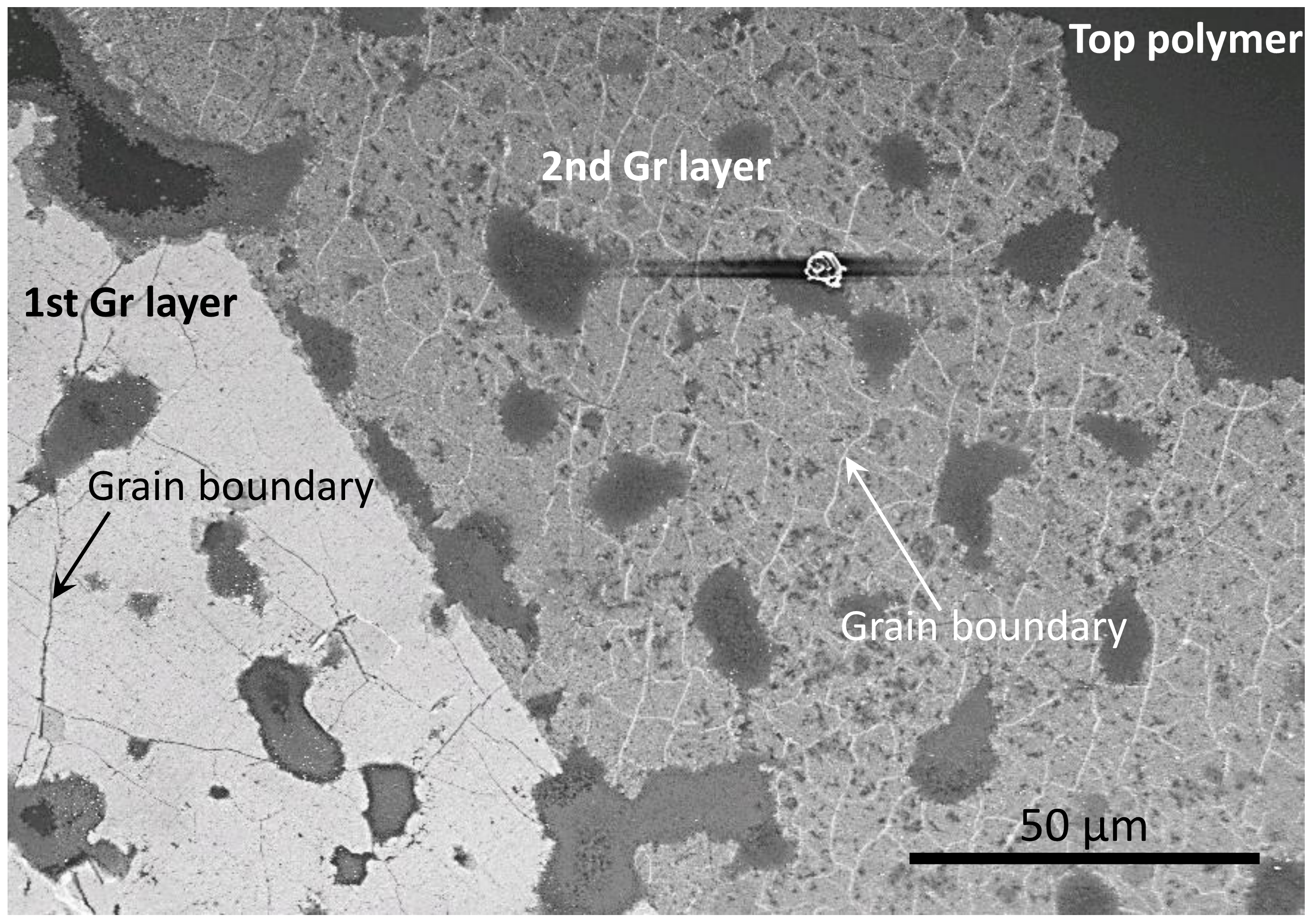


**Figure S2:** SEM micrograph of the nanocomposite film shows individual graphene and polymer layers in the nanocomposite stacking. Clear grain boundaries were observed in first graphene layer. These grain boundaries react as scattering center during carrier conduction and reduce sheet resistance as demonstrated earlier. Second graphene layer transferred on top could improve carrier conduction by opening new conduction paths through second layer however, it also contained grain boundaries as demonstrated. These grain boundaries were further covered by polymer layer on top. Random polymer spots observed outside of the polymer region could be formed during the spin coating process. The surface morphology the nanocomposite film after the formation of polymer layer was comparably smoother compare to graphene regions. This could beneficial for the surface roughness sensitive optoelectronic devices such as solar cells, light emitting, and electroluminescent devices, where smoother surface morphologies were critically important in FTCF to reduce surface induced carrier scattering and consequent performance degradations.


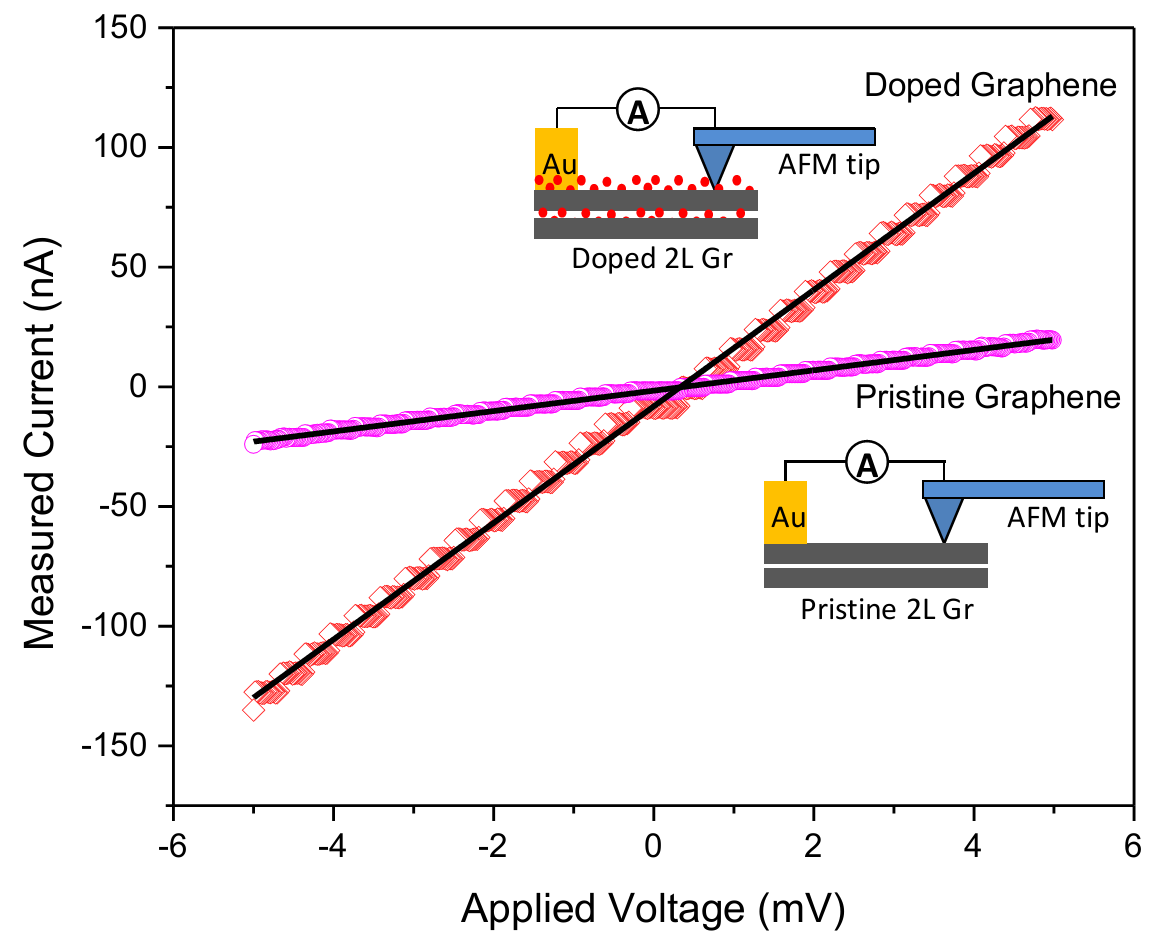


**Figure S3:** The current voltage characteristics measure between AFM tip and in contact film surface clearly demonstrates higher current flow in the DG compare to PG sample. This clearly suggest that surface conductance the graphene films can be increased by using mixed chemical doping method.


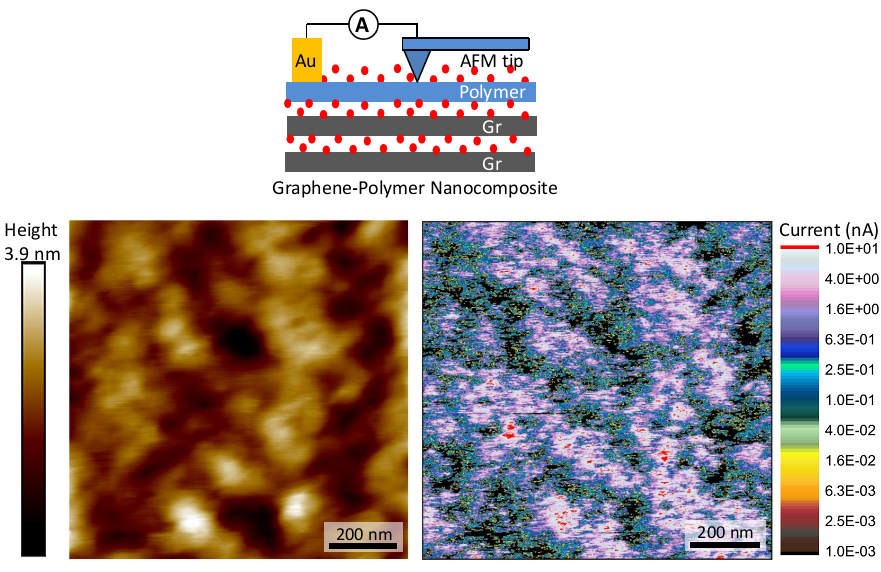


**Figure S4:** The surface conductance properties of the DGPN sample. In this case AFM tip force was restricted to 28.2 nN in order to prevent mechanical damage of the polymer layer induced by the AFM tip. AFM height profile map and surface current plot show measured current in the order of 4 nA range in DGPN sample.


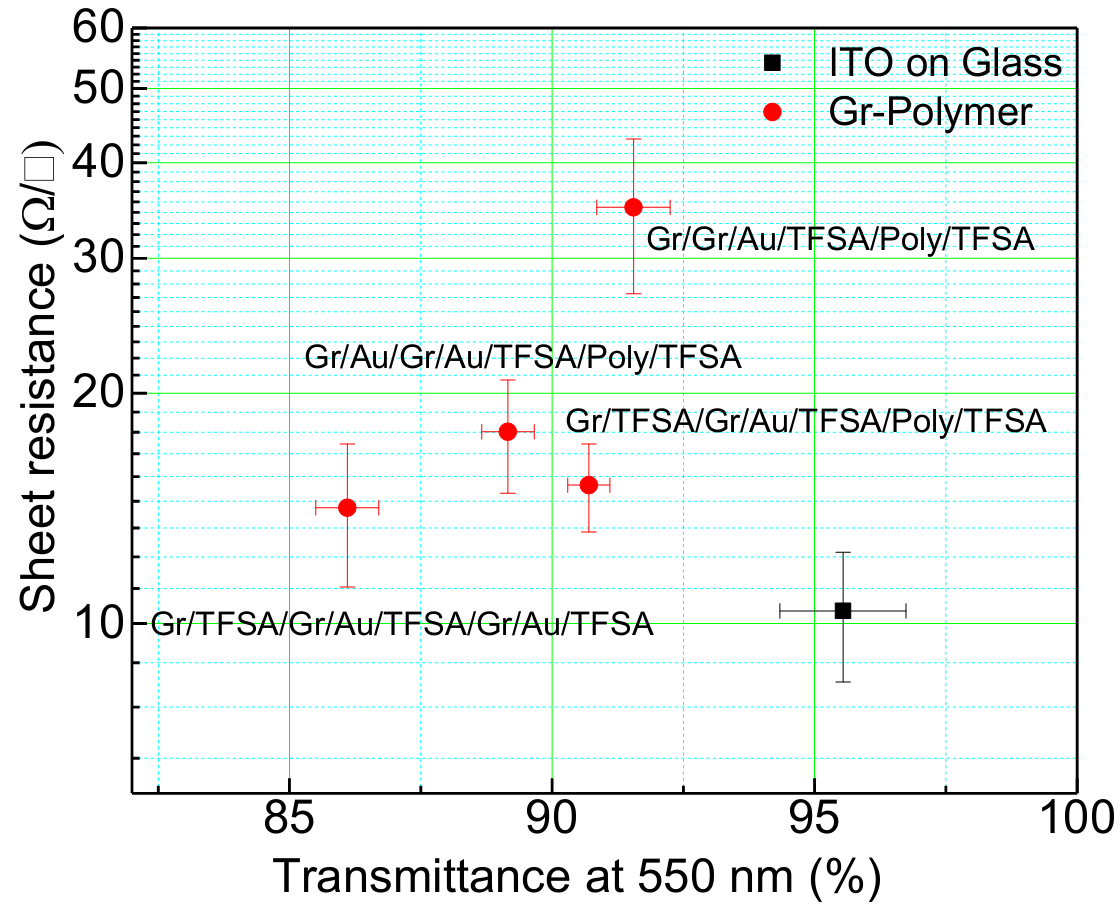


**Figure S5:** Statistical sample variations of transmittance and sheet resistance of Gr-polymer nanocomposite and ITO coated glass.
